# Renal Dysfunction after Living-Donor Liver Transplantation: Experience with 500 Cases

**DOI:** 10.1155/2018/5910372

**Published:** 2018-12-23

**Authors:** Ehab E. Abdel-Khalek, Alrefaey K. Alrefaey, Amr M. Yassen, Ahmed Monier, Hesham M. Elgouhari, Mohamed Samy Habl, Gehad Tawfik, Thuraya Elzayat, Reham Adly Zayed, Mohamed Abdel-Wahab

**Affiliations:** ^1^Liver Transplantation Unit and Department of Internal Medicine, Faculty of Medicine, University of Mansoura, Mansoura, Egypt; ^2^Department of Anaesthesia and Surgical Intensive Care, Faculty of Medicine, University of Mansoura, Mansoura, Egypt; ^3^Liver Transplantation Unit and Gastroenterology Center, Faculty of Medicine, University of Mansoura, Mansoura, Egypt; ^4^Department of Internal Medicine, Sanford School of Medicine, University of South Dakota, USA; ^5^House Officer, University of Mansoura, Mansoura, Egypt

## Abstract

*Introduction. *The possible risk factors for chronic kidney disease in transplant recipients have not been thoroughly investigated after living-donor liver transplantation.* Material and Methods. *A retrospective cohort study of consecutive adults who underwent living-donor liver transplantation between May 2004 and October 2016, in a single center, was conducted. Kidney function was investigated successively for all the patients throughout the study period, with 12 months being the shortest follow-up. Postoperative renal dysfunction was defined in accordance with the Chronic Kidney Disease Epidemiology Collaboration criteria. The patients' demographic data, preoperative and intraoperative parameters, and outcomes were recorded. A calcineurin inhibitor-based immunosuppressive regimen, either tacrolimus or cyclosporine, was used in all the patients.* Results. *Of the 413 patients included in the study, 33 (8%) who survived for ≥1 year experienced chronic kidney disease 1 year after living-donor liver transplantation. Twenty-seven variables were studied to compare between the patients with normal kidney functions and those who developed chronic kidney disease 1 year after living-donor liver transplantation. Univariate regression analysis for predicting the likelihood of chronic kidney disease at 1 year revealed that the following 4 variables were significant: operative time,* P *< 0.0005; intraoperative blood loss,* P *< 0.0005; preoperative renal impairment,* P* = 0.001; and graft-to-recipient weight ratio (as a negative predictor),* P *< 0.0005. In the multivariate regression analysis, only 2 variables remained as independent predictors of chronic kidney disease at 1 year, namely, operative time with a cutoff value of ≥714 minutes and graft-to-recipient weight ratio as a negative predictor with a cutoff value of <0.91.* Conclusion. *In this study, prolonged operative time and small graft-to-recipient weight ratio were independent predictors of chronic kidney disease at 1 year after living-donor liver transplantation.

## 1. Introduction

Liver transplantation (LT) was approved as a definitive therapy for end-stage liver disease outside the experimental realm by the United States National Institute of Health (USNIH) in 1983. Since then, LT has altered the natural history of end-stage liver disease and is now considered the accepted therapy for a wide spectrum of previously fatal liver diseases [1].

Serum bilirubin level, the international normalized ratio of prothrombin time, and serum creatinine level are the 3 components of the model for end-stage liver disease (MELD), which has served as the basis for liver allocation since February 2002. This has led to the expansion of the role of renal function assessment during the pretransplant evaluation and throughout the follow-up period [2].

In the literature, kidney function abnormalities before transplantation are mostly associated with a higher possibility of intraoperative complications, infection, prolonged postoperative hospital stay, need for dialysis, and overall financial burden [3]. Moreover, renal failure is associated with increased mortality of patients admitted in intensive care unit in general and in liver transplant recipients in particular, ranging between 27% and 67% depending on the comorbidities [4]. Gonwa et al. reported that 35% of liver transplant recipients with hepatorenal syndrome (HRS) and only 5% without HRS needed renal replacement therapy (RRT) postoperatively [5]. Renal function was not thoroughly studied after living-donor LT (LDLT).

The aim of this study was to assess the incidence and determine the possible risk factors of chronic kidney disease (CKD) in recipients 1 year after LDLT.

## 2. Materials and Methods

This was a single-center retrospective cohort study. The research protocol was reviewed and approved by the Institutional Research Board and Ethical Committee of the Faculty of Medicine, Mansoura University (Protocol No. R/16.12.82). All data were collected and analyzed to ensure data integrity and patient privacy. The study was conducted in the Gastroenterology Center, Mansoura University, Egypt.

Data of all the patients who underwent LDLT in Mansoura University Gastroenterology Center between May 2004 and October 2016 were collected from a prospectively maintained database. Data were analyzed to detect risk factors of kidney dysfunction after LDLT and its impact on 1-year graft and patient survival. The exclusion criteria were age of <18 years at the time of surgery, the need for preoperative renal replacement therapy (RRT), and/or death within the first 12 months of transplantation.

Patient selection, preoperative assessment, and perioperative management were performed by the same transplant team, including experienced hepatologists, surgeons, anesthetists, and radiologists. The preoperative data included the patients' demographics, MELD score, basal serum creatinine level, preoperative GFR, liver function tests, Child-Pugh classification, presence of ascites, serum electrolyte levels, and urine analysis. Routine renal function assessment before surgery included serum creatinine level, blood urea nitrogen level, serum uric acid level, urinalysis, and renal ultrasonography. A nephrological consultation was requested for cases with elevated serum creatinine levels of ≥1.5 mg/dl, proteinuria, evidence of acute kidney injury (AKI), or abnormal renal ultrasonographic findings. Intraoperative records were screened for blood loss, hypotensive events, urine output, graft-to-recipient weight ratio (GRWR), warm ischemia time, cold ischemia time, duration of surgery, and vascular complications.

The postoperative data included daily serum creatinine levels and examination for detection of possible postoperative events in form of sepsis, bleeding, bile leak, primary graft failure, delayed graft function, rejection, or ischemia-reperfusion injury. Kidney functions were assessed at the following time points: days 1, 2, 3, 7, and 14 after surgery, 1 month, 3 months, and 1 year after surgery. In case of diagnosed renal impairment, patients were subjected for further assessment.

We did not care much about the transient early postoperative fluctuations of renal function parameters which were corrected by manipulations of immunosuppressive drugs, antibiotics, and fluid balance. CKD was defined as an estimated GFR (eGFR) of <60 mL/(min·1.73 m^2^) for at least 3 months or >60 mL/(min·1.73 m^2^), with parameters denoting kidney damage for at least 3 months. Postoperative renal dysfunction was defined according to the Chronic Kidney Disease Epidemiology Collaboration criteria [6].

LDLT recipients were classified at the time of data analysis into 2 groups as follows, with the shortest follow-up being 12 months after the time of surgery: group I, with normal kidney functions, and group II, those who developed CKD.

Most of the recipients had hepatitis C virus (HCV) infection; thus, our program adopted a steroid-free protocol apart from an initial dose of methylprednisolone administrated intravenously (IV) at a dose of 10 mg/kg of the recipient's weight, immediately after reperfusion of the graft. In addition, IV infusion of 20 mg basiliximab was given on reperfusion and on the fourth postoperative day. A calcineurin inhibitor- (CNI-) based immunosuppressive regimen, either tacrolimus or cyclosporine, was used in all the patients. Tacrolimus therapy was started within the first 12 hours after reperfusion at an oral dosage of 2 × 0.05 mg/(kg·day). The tacrolimus dose was adjusted to a target range of 10-15 *μ*g/L during the first 3 months and 5-10 *μ*g/L after the third month. If cyclosporine was used, a dose of 4 mg/(kg·day), divided twice daily, was administered. The trough level was kept between 150 and 200 *μ*g/L in the first 6 months and then at 100-150 *μ*g/L. Mycophenolate mofetil was used as a part of the initial therapy or as a maintenance immunosuppressive agent. It was given orally starting from the first postoperative day at a dosage of 2 × 15 mg/(kg·day).

In cases with preoperative renal insufficiency (RI) or perioperative AKI, administration of CNIs was delayed for 72 hours after surgery and then a lower target level was adopted (5-10 *μ*g/L) [7].

Data were entered and analyzed using the IBM-SPSS version 21 software. Categorical data were expressed as number (percentage) and compared using the chi-square test (or Fisher exact test). Quantitative data were initially tested for normality by using the Kolmogorov-Smirnov test, where data were considered normally distributed if the* P* value was >0.050. Quantitative data were expressed as mean ± SD and compared between two groups by using the independent-samples* t* test if normally distributed or as median (interquartile range [IQR]) or the Mann-Whitney* U* test if not normally distributed. The receiver-operating characteristic (ROC) curve was plotted between the “sensitivity” (true positive rate) and “1- specificity” (false-positive rate) across a series of cutoff points. The area under the ROC curve is considered as an effective measure of inherent validity of a diagnostic test. This curve is useful in finding the optimal cutoff point to minimize misclassification of diseased and nondiseased subjects. Predicting the likelihood of a dichotomous variable was performed using a logistic regression analysis.

## 3. Results

During the study period, 500 patients underwent LDLT at the Liver Transplantation Unit, Gastroenterology Center, Mansoura University, Egypt. Their mean age was 51 years (range, 10–64 years). Most of the recipients were men (446, 89.2%). Their median MELD score was 15 (range, 6–48). Most of our patients had chronic HCV infection (453, 90.6%), which was the main indication for LDLT in our study (323, 64.6%). The patients' demographics are shown in Table 1.

**Figure 1:**
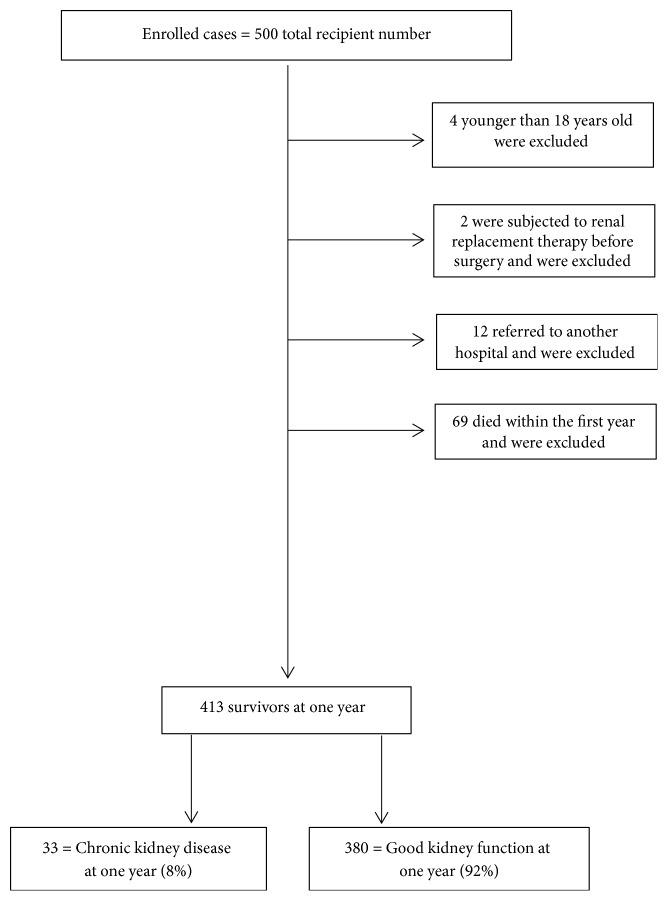
Flowchart.

We excluded 87 patients because they were aged ≤18 years (n = 4), were subjected to renal replacement therapy before surgery (n = 2), had no complete follow-up after transplantation or had been referred to another hospital (n = 12), or died during the first 12 months after surgery (n = 69). Of the 413 patients, 33 (8%) developed CKD at 1 year after LDLT (Figure 1).

The comparison between group I patients with normal kidney functions and group II patients who developed CKD 1 year after LT is shown in Table 2.

**Figure 2:**
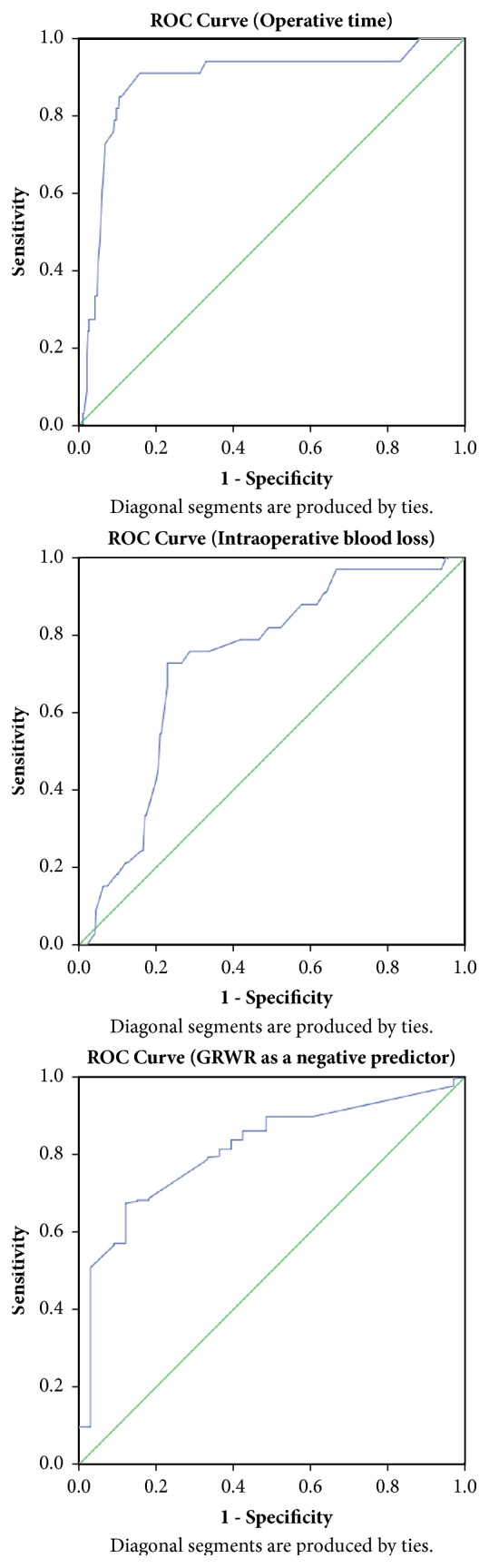
ROC curve.

Cutoff values of ≥ 714 minutes for the operative time, ≥ 7750 ml for the blood loss and ≥ 0.91 for graft-to-recipient weight ratio as a negative predictor were calculated by the receiver-operating characteristic (ROC) curve analysis (Table 3 and Figure 2) to be used in the univariate regression analysis for predicting the likelihood of chronic kidney disease at one year (Table 4).

*Multivariate Regression Analysis for Predicting the Likelihood of CKD at 1 Year*. A binomial logistic regression was performed to ascertain the effects of preoperative RI, operative time (≥714 minutes), blood loss (≥7750 ml), and GRWR (<0.91, as a negative predictor) on the likelihood that participants would have CKD at 1 year. One studentized residual had a value of 2.649 standard deviations, and two studentized residuals had a value of −2.749 standard deviations, which were kept in the analysis.

The logistic regression model was statistically significant,* χ*^2^(4) = 119.599,* P* < 0.0005. The model explained that 58.9% (Nagelkerke* R*^2^) of the variance in RI at 1 year and correctly classified 95.4% of cases. The sensitivity was 97.6%; specificity, 69.7%; positive predictive value, 71.9%; and negative predictive value, 97.4%.

Of the 4 predictive variables, operative time (≥714 minutes) and GRWR (<0.91, as a negative predictor) were statistically significant (as shown in Table 5). The patients with operative times of ≥714 minutes had 37.7 times higher odds to exhibit CKD at 1 year, whereas the patients with a GRWR of <0.91 (as a negative predictor) had 0.072 times higher odds to exhibit RI at 1 year, which means that they had 13.9 times higher odds to not exhibit CKD at 1 year (negative predictor).

## 4. Discussion

LT has become the only option for patients with end-stage liver disease, and this procedure is permitted in our country from only living donors because deceased donation still lacks legislation [8]. CKD remains a common disorder after LT in spite of the progress of preoperative evaluation, anesthetic medications, surgical techniques, postoperative care, and immunosuppressive therapy. The rate of renal dysfunction is varied among different studies in the context of LT in relation to the source of the graft, whether living or deceased donors; the timing of kidney function monitoring; and the different definitions used for kidney dysfunction [9–12]. Thus, these definitions should be standardized for a better comparison of studies worldwide. We found that approximately 8% of LDLT recipients have some degree of CKD by the first postoperative year. This rate varies among different studies. Barreto et al. observed a 47% prevalence of some degree of kidney dysfunction [13]. Another 2 studies found a prevalence of approximately 30% [9, 14]. A third group of researchers found only 12% of patients developing renal dysfunction after orthotopic LT [15]. The aim of this study was to clarify the occurrence of renal dysfunction at the point of one year after surgery rather than the time of onset. It was a time point not a time-to-event (survival analysis). To the best of our knowledge, the lower prevalence in this study may be related to the living donation, a result shared by Atalan et al. [16].

In the present study, preoperative renal impairment was significantly more prevalent in the group II patients who developed CKD 1 year after surgery than in group I. This observation has also been reported by previous studies [9, 10, 14, 15, 17]. This may be explained by the following multiple factors: First, preoperative hemodynamic changes may enhance the risk of renal dysfunction in cases of liver cirrhosis by impairment of renal perfusion through immune-mediated vasodilatation, parietal and renal parenchymal edema, hypoalbuminemia, and renin-angiotensin-aldosterone axis disturbances that lead to intravascular hypovolemia. Second is the delay of biological markers uncovering severe renal damage [18–20]. Third, intrinsic CKD predisposes patients with end-stage liver disease to kidney dysfunction, stressing on the evident link between the effect of the 2 systems manifested by the more severe encephalopathy, shock, and the higher international normalized ratios in the patients with severe renal dysfunction than in the patients without renal dysfunction [18–21]. In spite of the significant difference of preoperative renal impairment between the 2 groups being a potential predictor of CKD at one year (crude odds ratio = 6.274), it was mild in all cases and related to liver disease as well as it was corrected before the time of surgery. Accordingly, in multivariate logistic regression analysis it was not found to be an independent predictor for the likelihood of CKD at one year and only prolonged operative time and graft-to-recipient weight ratio were the only independent predictors of CKD at 1 year. Owing to our long-term aims, we did not take into account the early postoperative transient changes of renal function parameters that were normalized by fluid correction and minor manipulations of antibiotics and immunosuppressive drugs without persistent effects.

Deceased liver donation is not legalized in our country till now, and only LDLT is allowed. This technique results in a partial graft that has a reduced overall parenchymal mass as compared with the whole-organ allograft. Such smaller grafts may be unable to meet the metabolic and hemodynamic demands of the recipients and may be implicated as a cause of allograft dysfunction and complications, including renal dysfunction, with a lower GRWR. Although GRWR of ≥0.8% is universally accepted, its median value (IQR 25–75) was 0.805 (0.8–0.9) in our group of patients who experienced renal dysfunction at 1 year as compared with 1.008 (0.9–1.2) in the other group, reaching a statistical significance (P < 0.0005). This finding is in accordance with a Korean study that included 284 cases of LDLT [22]. This may be explained as part of small-for-size (SFS) graft syndrome with poorly defined pathogenesis representing a group of manifestations and complications of insufficient graft size or function without an obvious technical explanation [23, 24]. We think that this is the first study that measured a cutoff GRWR of <0.91 as a predictor of renal dysfunction 1 year after LT.

We observed that the operative time was more prolonged in the renal impairment group than in the healthy group, with a statistically significant difference. A logical explanation would be the relationship between surgical duration and clamping with frequent hypotensive episodes and the need for more norepinephrine and the use of fluids rich in chloride. This finding is in agreement with those reported by relatively recent studies [25–30]. We analyzed the data, which resulted in an unpreceded cutoff time of ≥714 minutes as a predictor of renal dysfunction 1 year after LT.

In our study, the median (IQR 25–75) intraoperative blood loss volume was 8500 cc (7000–9750) in our group of patients who experienced renal dysfunction at 1 year as compared with 5400 cc (3700–7600 cc) in the other group, reaching statistical significance (P < 0.0005) [27]. This finding was supported by the fact that meticulous control of bleeding intraoperatively and stabilization of hemodynamic and electrolyte disturbances with correction of myocardial functions are crucial for prevention of renal disturbances after LT [11].

In addition, we did not observe a significant difference between the 2 groups in relation to the immunosuppression protocol, in contrast to other studies [16, 17]. In our center, the immunosuppressive protocol adopts a low-dose tacrolimus therapy (serum target of 5–8 ng/mL) with mycophenolic acid, which corresponds to the renal function-sparing immunosuppression regimen in many studies [31–34]. This protocol could significantly minimize kidney dysfunction in comparison with the standard immunosuppressant treatment with CNI (serum tacrolimus target of 8–10 ng/mL) [16, 17].

In conclusion, our study demonstrated that preliminary data revealed that 4 factors predicted the occurrence of CKD 1 year after LDLT. These factors are preoperative renal impairment, GRWR, intraoperative blood loss, and operative time. In the multivariate regression analysis, only 2 factors remained as independent predictors of CKD at 1 year, namely, GRWR as a negative predictor with a cutoff value of <0.91 and prolonged operative time with a cutoff value of ≥714 minutes. The retrospective design of this study is an important limitation because of the possibility of missing data. Further larger studies are recommended to better understand the tolerability and safety of different immunosuppression protocols on the pathophysiology of graft-associated renal dysfunction with the possible factors related to poor outcome.

## Figures and Tables

**Table 1:** Demographic data of the study cases.

Variable	All cases (n = 500)
Age, years	51 (10-64)
Gender	
Male	446 (89.2%)
Female	54 (10.8%)
Body mass index (kg/m^2^)	28.6 (16.7-42.5)
Child-Pugh score	9 (5-15)
MELD score	15 (6-48)
Virology	
HCV	453 (90.6%)
HBV	8 (1.6%)
Blood group	
A –ve	12 (2.4%)
A +ve	166 (33.2%)
AB –ve	3 (0.6%)
AB +ve	36 (7.2%)
B –ve	6 (1.2%)
B +ve	116 (23.2%)
O –ve	16 (3.2%)
O +ve	131 (26.2%)
Waiting period	5.5 (1-50)
Indication	
HCV	323 (64.6%)
HBV	6 (1.2%)
HCV + HBV	2 (0.4%)
HCC	146 (29.2%)
BCS	6 (1.2%)
Autoimmune	11 (2.2%)
Cryptogenic	5 (1%)
Sclerosing cholangitis	1 (0.2%)

MELD, model for end stage liver disease; HCV, hepatitis C virus; HBV, hepatitis B virus; HCC, hepatocellular carcinoma; BCS, Budd Chiari syndrome.

**Table 2:** Profile of both groups at one year after liver transplantation.

Variable	Normal kidney function at one year	Chronic kidney disease at one year	*P *Value
	Group I (n = 380)	Group II (n = 33)	
Age, years*∗*	51 (46-55) (24-63)	49 (45.5-55) (43-63)	0.642
Gender male:female, n	341:39	30:3	0.831
Body mass index, kg/m^2^*∗*	28.4 (25.6-31.1) (17.6-43.74)	27.9 (26.0-31.2) (21.3-34.63)	0.807
Serum creatinine before Tx*∗*	0.8 (0.6-1.0) (0.5-2.2)	0.7 (0.6-0.9) (0.5-1.2)	0.197
Serum albumin before Tx, g/dl*∗*	2.9 (2.5-3.3) (1.5-5.5)	2.7 (2.5-3.1) (1.5-3.9)	0.237
Serum total bilirubin before Tx, mg/dl*∗*	2.9 (1.8-4.3) (0.3-25)	2.6 (1.65-4.35) (0.5-10.5)	0.741
INR before Tx*∗*	1.6 (1.3-1.9) (1-4.9)	1.5 (1.35-2.1) (1.1-3.19)	0.951
MELD score before Tx*∗*	15 (13-18) (6-40)	16 (12.5-20) (9-34)	0.291
GRWR*∗*	1.008 (0.9-1.2) (0.77-1.8)	0.805 (0.8-0.9) (0.77-1.35)	**< 0.0005**
Preoperative renal impairment, n (%)	13 (3.42105)	6 (18.18181)	**< 0.0005**
HRS before Tx, n (%)	12 (3.2)	5 (15.2)	**0.001**
CI, minutes*∗*	35 (23-50) (10-175)	35 (25.5-54.5) (16-120)	0.349
WI, minutes*∗*	44.5 (34-66.75) (18-175)	49 (40-65) (31-95)	0.306
Hepatic vein anastomoses, n (%)			
1	258 (67.9)	18 (54.5)	
2	70 (18.4)	10 (30.3)	0.267
3	37 (9.7)	5 (15.2)	
4	14 (3.7)	0 (0.0)	
5	1 (0.3)	0 (0.0)	
Portal vein anastomoses, n (%)			
1	372 (97.9)	33 (100.0)	0.400
2	8 (2.1)	0 (0.0)	
Hepatic artery anastomoses, n (%)			
1	371 (97.6)	33 (100.0)	0.371
2	9 (2.4)	0 (0.0)	
Intraoperative blood loss, ml*∗*	5400 (3700-7600) (550-16000)	8500 (7000-9750) (1700-14000)	**< 0.0005**
Fresh frozen plasma, n (%)			
0	235 (61.8)	14 (42.4)	
1-6 units	48 (12.6)	5 (15.2)	0.072
More than 6 units	97 (25.5)	14 (42.4)	
Operative time, minutes*∗*	575 (430-660) (300-960)	800 (775-855) (400-940)	**< 0.0005**
Immunosuppression in ICU			
Tacrolimus	244 (64.2)	20 (60.6)	
Cyclosporin	85 (22.4)	8 (24.2)	0.916
Others	51 (13.4)	5 (15.2)	
AKI in ICU by creatinine criteria, n (%)	119 (31.315789)	9 (27.27272)	0.630
AKI in ICU by urine output criteria, n (%)	44 (11.57894)	5 (15.15151)	0.543
Biliary complications, n (%)	28 (7.4)	2 (6.1)	0.781
Acute rejection, n (%)	37 (9.7)	2 (6.1)	0.488
Hemodialysis, n (%)	3 (0.8)	0 (0)	0.608
Renal impairment at one month, n (%)	70 (18.4)	8 (24.2)	0.412
Survival, months*∗*	70 (45.25-96) (12-156)	70 (52.5-98) (12-156)	0.358

**∗**Data were expressed as median (interquartile range, IQR 25-75) (minimum-maximum) unless otherwise stated.

Statistically significant values are in bold.

Tx, transplantation; INR, international normalized ratio; MELD, model for end stage liver disease; GRWR, graft-to-recipient weight ratio; HRS, hepatorenal syndrome; CI, cold ischemia; WI, warm ischemia; ICU, intensive care unit; AKI, acute kidney injury.

**Table 3:** Accuracy of operative time, blood loss, and GRWR in predicting renal impairment at one year after liver transplantation.

**Variable**	**Cutoff value**	**AUC (95**% **CI)**	*P* **Value**	**Sensitivity**	**Specificity**	**PPV**	**NPV**
Operative time (minutes)	≥ 714	0.888 (0.819-0.958)	**< 0.0005**	90.9%	84.2%	33.3%	99.1%
Blood loss (ml)	≥ 7750	0.730 (0.652 – 0.809)	**< 0.0005**	72.7%	77.1%	21.6%	97.0%
GRWR (negative predictor)	≥ 0.91	0.811 (0.745 – 0.878)	**< 0.0005**	32.6%	93.5%	81.0%	01.5%

Statistically significant values are in bold.

AUC, area under curve; PPV, positive predictive value; NPV, negative predictive value; GRWR, graft-to-recipient weight ratio.

**Table 4:** Univariate regression analysis for predicting the likelihood of chronic kidney disease at one year.

Variable	CKD group		Chi-Square test		Standard Logistic regression	
	NO CKD	CKD	*X* ^2^	P	Crude OR	P
	(n=380)	(n=33)			(95% CI)	
Operative time:						
<714 min	320 (%)	3 (%)	100.533	**<0.0005**	—	**<0.0005**
≥714 min	60 (%)	30 (%)			53.3	
Blood loss:						
<7750 ml	293	9	38.366	**<0.0005**	—	**<0.0005**
≥7750 ml	87	24			8.98	
Preoperative RI						
Absent	367	27	15.074	**<0.0005**	—	**0.001**
Present	13	6			6.274	
GRWR:						
<0.91	256	4	39.738	**<0.0005**	—	**<0.0005**
≥0.91	124	29			0.067	

CKD, chronic kidney disease; OR, odds ratio; RI, renal impairment; GRWR, graft-to-recipient weight ratio.

**Table 5:** Multivariate regression analysis for predicting the likelihood of chronic kidney disease at one year.

Variable	B	SE	Wald	P	OR (95% CI)
Operative time (≥714 min)	3.630	0.676	28.804	**<0.0005**	37.694 (10.015-141.879)
Blood loss (≥7750 ml)	0.852	0.532	2.561	0.110	2.344 (0.826-6.653)
Preoperative RI	1.012	0.886	1.303	0.254	2.751 (0.484-15.632)
GRWR (≥0.91)	-2.634	0.601	19.233	**<0.0005**	0.072 (0.022-0.233)
Constant	-3.951				

OR, odds ratio; RI, renal impairment; GRWR, graft-to-recipient weight ratio.

## Data Availability

The data used to support the findings of this study are available from the corresponding author upon request.
